# Long-term mortality in older patients discharged after acute decompensated heart failure: a prospective cohort study

**DOI:** 10.1186/s12877-017-0419-2

**Published:** 2017-01-26

**Authors:** Pierre-André Natella, Philippe Le Corvoisier, Elena Paillaud, Bertrand Renaud, Isabelle Mahé, Jean-François Bergmann, Hervé Perchet, Dominique Mottier, Olivier Montagne, Sylvie Bastuji-Garin

**Affiliations:** 1 Université Paris Est (UPEC), A-TVB DHU, IMRB, EA7376, CEpiA Clinical Epidemiology and Ageing unit, Créteil, France; 2 AP-HP, Hôpital Henri Mondor, Service de Santé Publique, Créteil, France; 3 Inserm, Centre d’Investigation Clinique, 1430 Créteil, France; 4 AP-HP, Hôpital Henri Mondor, Pôle Vigilance Recherche Méthodologie & Information Médicale, 94010 Créteil, France; 5 AP-HP, Hôpital Henri Mondor, Département de Médecine Interne et Gériatrie, Créteil, France; 6 AP-HP, Hôpital Henri Mondor, Structure des Urgences, Créteil, France; 7 AP-HP, Hôpital Lariboisière-Fernand Widal, Département de Médecine Interne, Paris, France; 8 Université Paris 7, EA REMES Université Paris Diderot, Sorbonne Paris Cité, Paris, France; 9 Centre hospitalier de Meaux, Service de Cardiologie, Meaux, France; 10 CHU Brest, Hôpital Cavale Blanche, Département de Médecine Interne et de Pneumologie, Brest, France; 11 Université de Bretagne Occidentale, EA 3878 (GETBO), Brest, France; 12 AP-HP, Hôpital Henri Mondor, Unité de Recherche Clinique, Créteil, France

**Keywords:** Acute decompensated heart failure, Long-term mortality, Elderly

## Abstract

**Background:**

Data are available on short- and intermediate-term mortality rates after discharge for acutely decompensated heart failure (ADHF). However, few studies specifically addressed ADHF outcomes in patients aged 75 years or over, who contribute more than half of all ADHF admissions. Our objectives here were to estimate the long-term mortality of patients aged 75 years or over who were discharged after admission for ADHF and to identify factors, especially geriatric findings, independently associated with 2-year mortality.

**Methods:**

This prospective cohort study in five French hospitals included consecutive patients aged 75 years or older and discharged after emergency-department admission for ADHF meeting Framingham criteria (*N =* 478; median age, 85 years; 68% female). Kaplan-Meier 1-year and 2-year survival curves were plotted. Admission characteristics independently associated with overall 2-year mortality were identified using multivariable Cox proportional-hazards regression.

**Results:**

Mortality was 41.7% (95% confidence interval [95% CI], 37.2%–53.5%) after 1 year and 56.0% (95% CI, 51.5%–60.7%) after 2 years. By multivariable analysis, independent predictors of 2-year mortality were male sex (hazard ratio [HR], 1.36; 95% CI, 1.00–1.82), age >85 years (HR, 1.57; 95% CI, 1.19–2.07), higher number of impaired activities of daily living (HR, 1.11 per impaired item; 95% CI, 1.05–1.17), recent weight loss (HR, 1.61; 95% CI, 1.14–2.28), and lower systolic blood pressure (HR, 0.86 per standard deviation increase; 95% CI, 0.74–0.99). Creatinine clearance ≤30 mL/min showed a trend toward an association with 2-year mortality (HR, 1.36; 95% CI, 0.97–2.00).

**Conclusion:**

Functional impairment before admission is associated with higher long-term mortality in patients ≥75 years admitted for ADHF. This study focused on geriatric markers not traditionally collected in heart-failure patients but did not analyse all cardiologic parameters associated with outcomes in other studies. Nevertheless, our findings may contribute to identify those patients admitted for ADHF who have the worst prognosis.

**Electronic supplementary material:**

The online version of this article (doi:10.1186/s12877-017-0419-2) contains supplementary material, which is available to authorized users.

## Background

Heart failure (HF) affects more than 15 million people in Europe, many of whom are elderly [1]. The heavy burden of morbidity and mortality associated with HF is comparable to that of many advanced malignancies [2]. The mean life expectancy of patients discharged after admission for HF has been estimated at 5.5 years [3]. Several studies investigated the short- and intermediate-term risk of death after discharge for acutely decompensated heart failure (ADHF). In patients aged 65 years or more, overall mortality ranged from 25% to 40% after 1 year [4–15] and from 22% to 52.9% after 2 years [16–18]. However, few studies specifically addressed ADHF outcomes in patients aged 75 years or over [9, 10], who contribute more than half of all patients admitted for ADHF [19]. Moreover, only two of these studies reported the associations linking geriatric syndromes to 1-year mortality in elderly patients with HF [6, 16], and none investigated clinical and laboratory variables concomitantly with geriatric findings. These knowledge gaps need to be filled, as the prevalence of chronic diseases increases with age, so that most patients older than 75 years have multiple co-morbidities [20]*.* We previously reported co-morbidities and functional impairments independently associated with in-hospital mortality of older patients admitted for ADHF (ELISA survey) [21]. Better knowledge of risk factors for long-term mortality may help to define follow-up and management goals and may improve treatment decision-making.

Here, our primary objective was to assess the long-term survival of patients aged 75 years or over and discharged after in-patient treatment of ADHF. We also aimed to identify factors, especially geriatric findings, independently associated with 2-year mortality. To achieve these objectives, we used data from the previously described ELISA cohort study of severe heart failure designed to identify patients with the worst prognosis as early as possible during their hospital stay [21].

## Methods

### Study design

ELISA is a prospective longitudinal cohort study of 680 older patients with ADHF seen at the emergency departments of five French hospitals [21], between October 2004 and December 2007. The ELISA cohort was established in compliance with good clinical practice guidelines and was approved by the appropriate ethics committee (institutional review board of the Henri-Mondor Teaching Hospital, Créteil, France). Cohort participants or their relatives gave their written informed consent for the collection of personal data to be used in further analyses. The present report complies with the Strengthening the Reporting of Observational Studies in Epidemiology (STROBE) statement [22].

We estimated 1-year and 2-year mortality rates and we looked for associations linking admission characteristics, including geriatric findings and co-morbidities, to 2-year mortality among hospital survivors.

### Patients

As previously described [21], consecutive patients aged 75 years or older and admitted to an emergency department with a diagnosis of ADHF during a 1-year study period were included in the ELISA cohort if they met Framingham criteria for HF (at least two major criteria or one major criterion plus two minor criteria) [23]; dyspnoea at rest or with minimal exertion; and an expected hospital stay duration ≥24 h. Exclusion criteria were ventricular arrhythmia at admission and transfer to another hospital after the initial evaluation. For the present study focusing on survival after hospital discharge, we excluded the patients who died in the hospital.

### Data collection

Included patients underwent a standardised clinical evaluation at the emergency department. However, some parameters such as the get-up-and-go test were collected as early as possible after the relief of ADHF symptoms. Baseline data included socio-demographic characteristics, medical history, clinical characteristics, and laboratory test results. Trained clinical research assistants recorded geriatric parameters using validated tools. Nutritional status was assessed using the Mini Nutritional Assessment-Short Form [24] to classify patients into three nutritional risk categories (≥12, well-nourished; 8-11, at risk; and <8, malnourished) [25]. Weight loss over the last three months (none, 1 to 3 kg, or >3 kg) was recorded from the patients or relatives then validated by medical record review. For cognitive status, assessed using the MMSE, the cut-off of 17 indicating severe cognitive impairment was chosen [26–28]. Mood was assessed using the 15-item Geriatric Depression Scale (GDS), with scores ≥5 indicating a risk of depression [29, 30]. Functional status was assessed using the Katz activities of daily living scale (ADL) with six items (bathing, dressing, toileting, transferring, continence, and feeding) scored from 2 (able to perform the activity) to 0 (unable to perform the activity) [31]. The number of impaired ADL items was recorded, as well as the presence of functional impairment defined as a need for assistance for at least one ADL (i.e., ADL score <12). Impaired mobility was defined as a get-up-and-go test time >20 s or an inability to perform the test [32]. Glomerular filtration rate was calculated using the abbreviated Modification of Diet in Renal Disease.

### Follow-up after hospital discharge

Patients were monitored by telephone calls at three-month intervals for 2 years after discharge or until death. Vital status was collected from the next of kin, usual physician, or patient’s residential institution. The primary outcome for the present analysis was overall 2-year mortality after discharge.

### Statistical analysis

Sample size was estimated for the main objective of the ELISA cohort, namely, the identification of factors associated with in-hospital mortality [21].

All tests were two-sided, and *P* values ≤0.05 were considered significant. Analyses were performed using STATA software version 12.0 (StataCorp, College Station, TX, USA). No imputation for missing data was performed.

Quantitative data are described as mean ± SD or median [25^th^-75^th^ centiles], as appropriate, and categorical variables as n (%). Overall survival was assessed from discharge to death from any cause, 2 years after hospital discharge or last follow-up for censored patients. We used the non-parametric Kaplan-Meier method to estimate 1-year and 2-year survival rates with their 95% confidence intervals (95% CIs).

Characteristics of survivors and non-survivors were compared using Cox proportional hazards regression models with estimation of hazard ratios (HRs) and their 95% CIs. Log-linearity was tested for quantitative variables. Analyses were routinely adjusted for age dichotomised based on the median value (>85 versus ≤85 years). Variables associated with *P* values <0.15 were selected for multivariable analyses. Confounders and interactions were tested in bivariate models. We also investigated a potential centre effect. To avoid introducing correlated variables into multivariable models, correlations were assessed using Cramer’s V for categorical variables [33] and Spearman’s non-parametric rank correlation (Rho) [34] for quantitative variables; values above 0.50 were considered to indicate correlations. The most relevant variables were identified based on clinical relevance, number of missing values, and the Akaike information criterion (AIC) [35, 36]. The proportional hazards assumption was assessed both graphically and statistically using the Schoenfeld residuals test. This assumption was met for all variables in the final models. Calibration and discrimination of the final multivariable model were assessed using the Gronnesby-Borgan goodness-of-fit test and Harrell’s c-index, respectively [37, 38]. Model robustness was tested using a non-parametric bootstrap resampling procedure. The final Cox model was refit for the 500 bootstrap samples thus created [39].

## Results

**Fig. 1:**
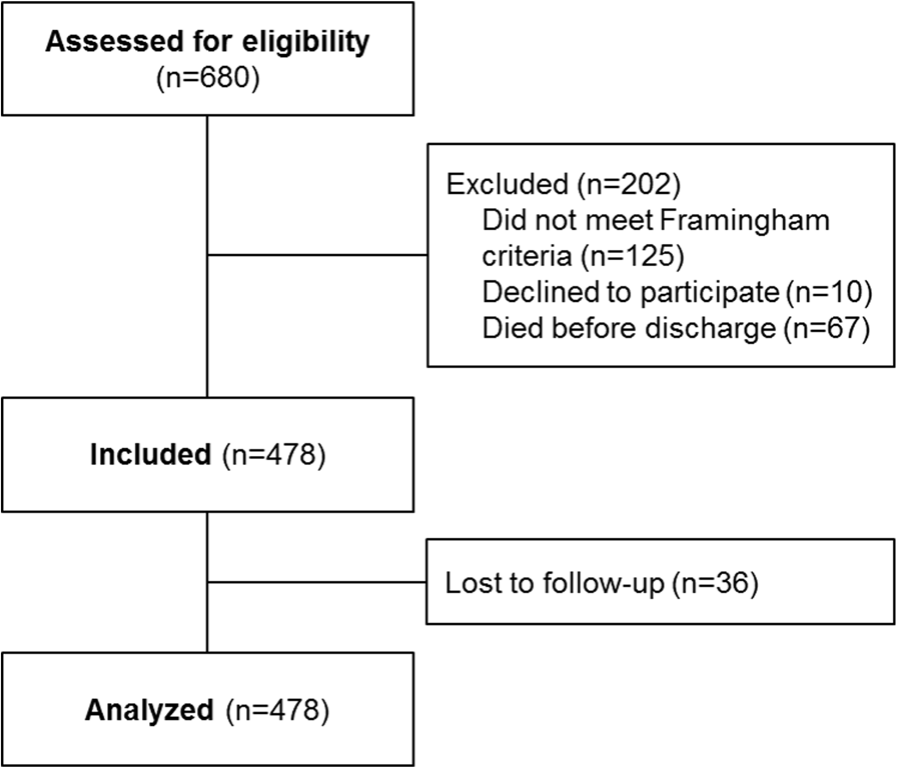
Participant flow chart

Among the 680 patients initially assessed for eligibility, 478 were analysed (Fig. 1). Table 1 reports their baseline characteristics. Median age was 85 years (range, 75-105 years). Most patients (91%) had one or more cardiovascular co-morbidities (myocardial infarction, stroke, hypertension, and/or arrhythmia). More than half of patients had abnormal nutritional status, function, and/or mobility. Polypharmacy was the rule (median, 7 drugs).

**Table 1:** Baseline characteristics of elderly patients admitted for acute decompensated heart failure

Characteristics	Total
	*n =* 478
Socio-demographic characteristics	
Age >85 years	222 (46)
Male sex	155 (32.4)
Living arrangements (*n =* 220/244)	
Lives alone	386 (83.2)
Institutionalised	(16.8)
Current or former smoker (*n =* 211/242)	110 (24.3)
Medical status	
Co-morbidities (past or current):	
Arteritis (*n =* 207/234)	53 (12.0)
Myocardial infarction (*n =* 220/251)	97 (20.6)
Stroke (*n =* 218/245)	51 (11.0)
Anaemia (*n =* 209/233)	116 (26.2)
Hypertension^a^ (*n =* 223/254)	354 (74.2)
Cardiac arrhythmia (*n =* 215/245)	313 (68.0)
Diabetes (*n =* 218/253)	84 (17.8)
Systolic blood pressure, mmHg (*n =* 210/249)	146 [128–165]
Number of drugs	7 [5–9]
≥ 5 drugs per day	382 (80.0)
Minnesota Living with Heart Failure Questionnaire (*n =* 100/102)	
< 24 (good quality of life)	54 (26.7)
[24–45] (intermediate quality of life)	97 (48.0)
> 45 (poor quality of life)	51 (25.2)
Laboratory parameters at admission	
Sodium, mmol/L (*n =* 282/188)	138 [135–141]
Haemoglobin, g/dL (*n =* 212/247)	12.3 (11.1–13.6)
Creatinine clearance, mL/minute^b^ (*n =* 218/250)	
≥ 60	213 (45.5)
]30–60[	196 (41.9)
≤ 30	59 (12.6)
Nutritional parameters	
Body mass index, Kg/m^2^ (*n =* 146/166)	
< 19	10 (3.2)
[19–21 [	18 (5.8)
[21–23 [	39 (12.5)
≥ 23	245 (78.5)
MNA-SF score (*n =* 69/81)	
≥ 12 (well-nourished)	58 (38.7)
[8–12] (at risk)	73 (48.7)
< 8 (malnourished)	19 (12.7)
Recent weight loss >3 Kg (<3 months) (*n =* 201/226)	64 (15.0)
Non-solid nutrition (blended or minced) (*n =* 215/238)	82 (18.1)
Function and mobility	
Number of impaired ADL items (*n =* 201/226)	2 [0–5]
ADL score <12, (*n =* 201/226)	268 (62.8)
Timed get-up-and-go >20 s^c^, (*n =* 178/210)	296 (76.2)
Cognition	
MMSE ≤17, severe impairment (*n =* 221/251)	97 (20.6)
Depression	
GDS score ≥5 (*n =* 143/148)	142 (48.8)

Quantitative variables are expressed as median [25th - 75th centiles] and categorical variables as N (%)

(*n*= /) indicates the number of patients in each group in case of missing data

MNA-SF, Mini Nutritional Assessment-Short Form; ADL, activities of daily living scale; MMSE, Mini Mental State Examination; GDS, Geriatric Depression Scale

^a^Hypertension was defined as blood pressure ≥140/90 mmHg or treatment for hypertension

^b^Creatinine clearance was calculated using the abbreviated Modification of Diet in Renal Disease formula, glomerular filtration rate (mL/min/1.73 m^2^) = 186.3 × [creatinine (μmol/L) /88.4] ^-1.154^ × [Age (years)]^-0.203^ × 0.742 (if female) × 1.21 (if black)

^c^Timed Get-Up-and-Go test >20 s or patient unable to perform the test

**Fig. 2:**
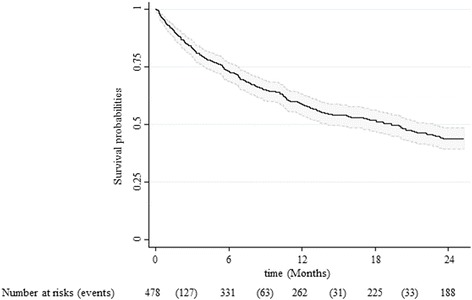
Kaplan-Meier survival distribution, with 95% confidence intervals, of 478 patients aged 75 years or over and discharged after admission for acutely decompensated heart failure

The 36 (7.5%) patients lost to follow-up were censored at the date of last information. Median follow-up was 14.1 months (449 days [132–718 days]; range, 1–770 days). Mortality was 41.7% (95% CI, 37.2%–53.5%) after 1 year and 56.0% (95% CI, 51.5%–60.7%) after 2 years (Fig. 2). Median overall survival was 19 months (1.6 years). Most deaths occurred early after hospital discharge, after a median of about 6 months (184 days [68–367 days]).

### Factors associated with 2-year survival

**Fig. 3:**
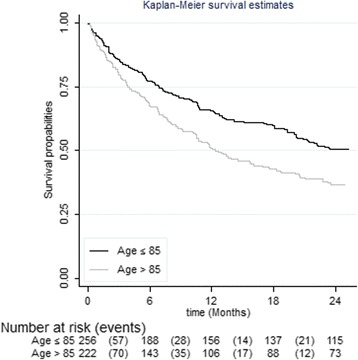
Kaplan-Meier survival distributions according to age

By univariate analysis (Table 2), factors significantly associated with mortality were older age (Fig. 3); male sex; anaemia; lower systolic blood pressure; renal failure; lower body mass index, malnutrition, and recent weight loss; and impairments in functional status and mobility. Trends (*P* < 0.15) were observed for history of myocardial infarction and severe cognitive impairment. Therefore, these variables were potential candidates for multivariable analysis. Neither other cardiovascular co-morbidities nor polypharmacy were associated with 2-year mortality. No centre effect or effect modification by centre was demonstrated.

**Table 2:** Comparison of survivors and non-survivors using age-adjusted Cox proportional hazards regression models

Characteristics	Survivors	Non-survivors	Age-adjusted analysis^a^	*P* value
	*n =* 224	*n =* 254	HR [95%CI]	
Socio-demographic characteristics				
Age >85 years	88 (39.0)	134 (53)	1.49 [1.17–1.91]	0.01
Male sex	63 (28.1)	92 (36.2)	1.32 [1.02–1.72]	0.03
Living arrangements (*n =* 220/244)				
Lives alone	188 (85.5)	198 (81.1)	1	
Institutionalised	32 (14.5)	46 (18.9)	1.17 [0.84–1.61]	0.35
Current or former smoker (*n =* 211/242)	53 (25.1)	57 (23.6)	1.00 [0.74–1.34]	0.99
Medical status				
Co-morbidities (past or current):				
Arteritis (*n =* 207/234)	19 (9.2)	34 (14.5)	1.28 [0.89–1.85]	0.18
Myocardial infarction (*n =* 220/251)	36 (16.4)	61 (24.3)	1.27 [0.95–1.69]	0.11
Stroke (*n =* 218/245)	20 (9.2)	31 (12.7)	1.29 [0.86–1.83]	0.24
Anaemia (*n =* 209/233)	44 (21.1)	72 (30.9)	1.34 [1.02–1.77]	0.04
Hypertension^b^ (*n =* 223/254)	166 (74.4)	188 (74.0)	0.96 [0.72–1.27]	0.76
Cardiac arrhythmia (*n =* 215/245)	141 (65.6)	172 (70.2)	1.02 [0.77–1.35]	0.87
Diabetes (*n =* 218/253)	43 (19.7)	41 (16.2)	0.93 [0.66–1.31]	0.70
Systolic blood pressure, mmHg (*n =* 210/249)^c^	150 [135–170]	141 [121–162]	0.83 [0.73–0.96]	0.01
Number of drugs	7 [5–9]	7 [5–9]		
≥ 5 drugs per day	171 (76.3)	211 (83.1)	1.26 [0.91–1.75]	0.17
Minnesota Living with Heart Failure Questionnaire (*n =* 100/102)				
< 24 (good quality of life)	29 (29.0)	25 (24.5)	1	0.69
[24–45] (intermediate quality of life)	46 (46.0)	51 (50.0)	1.16 [0.72–1.88]	
> 45 (poor quality of life)	25 (25.0)	26 (25.5)	1.27 [0.73–2.21]	
Laboratory parameters at admission				
Sodium, mmol/L (*n =* 282/188)	138 [135–141]	138 [135–141]	0.99 [0.97–1.01]	0.43
Haemoglobin, g/dL (*n =* 212/247)	12.5 [11.3–13.7]	12.1 [10.9–13.4]	0.97 [0.92–1.04]	0.40
Creatinine clearance, mL/minute^d^ (*n =* 218/250)				
≥ 60	117 (53.7)	96 (38.4)	1	
[30–60]	83 (38.1)	113 (45.2)	1.28 [0.97–1.68]	0.08
≤ 30	18 (8.3)	41 (16.4)	1.81 [1.25–2.61]	<0.01
Nutritional parameters				
Body mass index, Kg/m^2^ (*n =* 146/166)				
< 19	2 (1.4	8 (4.8)	2.30 [1.11–4.74]	0.03
[19–21[	5 (3.4)	13 (7.8)	1.50 [0.84–2.67]	0.19
[21–23[	14 (9.6)	25 (15.1)	1.50 [0.98–2.31]	0.08
≥ 23	125 (85.6)	120 (72.3)	1	
MNA-SF score (*n =* 69/81)				
≥ 12 (well-nourished)	33 (47.8)	25 (30.9)	1	
[8–12[(at risk)	31 (44.9)	42 (51.9)	1.64 [0.99–2.70]	0.05
< 8 (malnourished)	5 (7.2)	14 (17.3)	3.01 [1.52–5.97]	<0.01
Recent weight loss >3 Kg (<3 months) (*n =* 201/226)	22 (10.9)	42 (18.6)	1.59 [1.22–2.06]	<0.01
Non-solid nutrition (blended or minced) (*n =* 215/238)	31 (14.4)	51 (21.4)	1.60 [0.94–1.76]	0.11
Function and mobility				
Number of impaired ADL items (*n =* 201/226)^e^	1 [0–4]	2 [0–5]	1.10 [1.05–1.17]	<0.01
ADL score <12, (*n =* 201/226)	106 (52.7)	162 (71.7)	1.61 [1.20–2.16]	<0.01
Timed get-up-and-go >20 s^f^, (*n =* 178/210)	125 (70.2)	171 (81.4)	1.49 [1.05–2.11]	0.03
Cognition				
MMSE ≤17, severe impairment (*n =* 221/251)	38 (17.2)	59 (23.5)	1.26 [0.94–1.69]	0.12
Depression				
GDS score ≤5 (*n =* 143/148)	71 (49.7)	71 (48.0)	0.96 [0.70–1.33]	0.81

HR, hazards ratio; CI, confidence interval; MNA-SF, Mini Nutritional Assessment-Short Form; ADL, activities of daily living scale; MMSE, Mini Mental State Examination; GDS, Geriatric Depression Scale

^a^Hazards ratios and confidence intervals were estimated using Cox proportional models adjusted for age (≤85 years versus >85 years)

^b^Hypertension was defined as blood pressure ≥140/90 mmHg or treatment for hypertension

^c^Hazards ratios and confidence intervals per increase by 1 standard deviation

^d^Creatinine clearance was calculated using the abbreviated Modification of Diet in Renal Disease formula, glomerular filtration rate (mL/min/1.73 m^2^) = 186.3 × [creatinine (μmol/L) /88.4] ^-1.154^ × [Age (years)]^-0.203^ × 0.742 (if female) × 1.21 (if black)

^e^Hazards ratios and confidence intervals per each additional impaired ADL item

^f^Timed Get-Up-and-Go test >20 s or patient unable to perform the test

Age, sex, creatinine clearance, anaemia, systolic blood pressure, myocardial infarction, weight loss, body mass index, MNA-SF score, ADL score, Timed Get-Up-and-Go score, and MMSE score were available for the multivariable Cox model. Mobility impairment, lower body mass index, and MNA-SF category were not introduced into multivariable models, as they correlated with functional impairment and recent weight loss, respectively (correlation index >0.5, *P* < 0.05). Because of their association with other parameters, anaemia, myocardial infarction, and cognitive impairment were not independently associated with death in the multivariable model (*P* > 0.15). No significant interactions were found between variables associated with 2-year mortality. By multivariable analysis (Table 3), five factors were independently associated with 2-year mortality, namely, male sex, age older than 85 years, higher number of impaired ADL items, recent weight loss, and lower systolic blood pressure. A trend was noted for renal failure. The final model had good calibration (*P* value of the goodness-of-fit test >0.20) and acceptable discrimination (Harrell’s c-index, 0.64). The HRs estimated after bootstrap resampling were close to those of the original model, suggesting excellent internal validity. All five factors were also independently associated with 1-year mortality (Additional file 1: Table S1).

**Table 3:** Factors independently associated with 2-year mortality by multivariable analysis (*n =* 399)

Characteristics	Model development^a^		Parameter estimates after bootstrapping methods	
	HR 95% CI	*P* value	Mean HR 95% CI	*P* value
Male sex	1.36 [1.00–1.82]	0.05	1.36 [1.00–1.83]	0.05
Age >85 years	1.57 [1.19–2.07]	<0.01	1.58 [1.19–2.08]	<0.01
Number of impaired ADL^b^ items	1.11 [1.05–1.17]	<0.01	1.11 [1.04–1.18]	<0.01
Recent weight loss^c^	1.61 [1.14–2.28]	<0.01	1.61 [1.12–2.32]	0.01
Systolic blood pressure (mmHg)^d^	0.86 [0.74–0.99]	0.04	0.85 [0.73–1.00]	0.05
Creatinine clearance ≤30 mL/minute^e^	1.36 [0.97–2.00]	0.09	1.38 [0.89–2.08]	0.12

HR, hazards ratio; CI, confidence interval; ADL, activities of daily living scale

^a^Hazards ratios and confidence intervals were estimated using Cox proportional models simultaneously adjusted for all variables listed in the table

^b^per additional impaired ADL item

^c^>3 Kg within the 3 months preceding admission

^d^per increase by 1 standard deviation

^e^Creatinine clearance was calculated using the abbreviated Modification of Diet in Renal Disease formula, glomerular filtration rate (mL/min/1.73 m^2^) = 186.3 × [creatinine (μmol/L) /88.4] ^-1.154^ × [Age (years)]^-0.203^ × 0.742 (if female) × 1.21 (if black)

## Discussion

High 1-year and 2-year mortality rates of 41.7% and 56.0%, respectively, were documented in unselected elderly patients discharged alive after treatment for ADHF. Independent risk factors for death within 2 years were older age, male sex, prior functional impairment, low systolic blood pressure at admission, and recent weight loss. A trend was observed for renal failure.

One-year mortality rates close to 40% have been reported in subgroups of elderly patients in Canada, the United States, and Israel [5, 7, 10, 11, 14], in keeping with the 41.7% rate in our ELISA cohort. Other studies obtained variable results. In a Scottish study, 1-year mortality rates were 49% and 56% in the groups aged 75–84 years and >84 years, respectively [4]. In contrast, several studies from Spain, Canada, the United States, and Europe (EHFS II survey) found 1-year mortality rates of about 30% [6, 8, 9, 13]. These discrepancies may reflect differences in inclusion criteria regarding age, ADHF versus newly diagnosed HF, and presentation to the emergency department versus elsewhere. The 2-year mortality rate of 56% in our study is consistent with previous reports from Brazil and the United States [17, 18]. Conversely, an Italian study [16] estimated 2-year mortality at 22.9%. This difference may be ascribable to the younger mean age of 74 years and lesser severity of HF (NYHA class II).

We identified several admission characteristics that predicted 2-year mortality in elderly patients admitted for ADHF. The discrimination level of our final multivariable model (Harrell’s c-index, 0.64) suggests an influence on long-term mortality of additional variables not evaluated in our study. Indeed, other factors previously associated with increased mortality in patients with HF, e.g., type and duration of HF or medical therapy, may also have affected patient outcomes in our study. Three of the six parameters independently associated with 1-year and 2-year mortality, namely, lower systolic blood pressure, renal failure, and functional impairment before admission, were also associated with in-hospital mortality in the ELISA cohort [21].

Several studies previously documented an independent adverse effect of male sex and older age on mortality in elderly patients with HF, in keeping with our results [4, 5, 7, 8, 11, 16]. Similarly, lower systolic blood pressure [9–11, 13, 16–18] and renal dysfunction [9–11] were also previously associated with mortality.

Few studies have assessed pre-admission functional impairment as a prognostic marker in elderly patients with ADHF. The association of this factor with 2-year mortality in our population is consistent with two studies assessing associations between findings from a global geriatric assessment and 1-year mortality [6, 12]. These two studies and ours produced similar results despite using different functional assessment tools (Katz ADL, Barthel index, or instrumental ADL), a fact that supports the validity of our results. That neither of the two previous studies found associations of mortality with other factors, including older age, male sex, systolic blood pressure, and renal impairment, may be ascribable to limited statistical power (88 and 162 patients). Furthermore, in the octogenarians of the EHFS II survey, disability (‘self-care problems’) independently predicted long-term mortality in the multivariable analysis [9]. Our results are consistent with those of studies in other clinical settings, in which functional status predicted mortality independently from the underlying medical conditions. Interestingly, the 2-year risk of death in our population increased by 11% for each additional impaired ADL item.

Another important finding from our study is the influence of nutritional status. Not only recent weight loss, but also malnutrition or risk of malnutrition, predicted mortality independently from age. The only previous study assessing the prognostic value of MNA results in elderly patients with HF found no association with mortality [6]. However, in older adults, malnutrition is often related to adverse health outcomes and strongly predicts mortality [40, 41]. Weight loss is common in end-stage HF and may reflect numerous mechanisms including neurohormonal dysregulation and an imbalance between anabolism and catabolism [42]. Our results show that recent weight loss is a major prognostic marker in elderly patients successfully treated for an episode of ADHF.

Two of the six factors independently associated with mortality are recognized indicators of fragility. Thus, recent weight loss is part of the Fried phenotype, and functional impairment is related to the slowness and low physical activity criteria [43]. Furthermore, impairment of one ADL item corresponds to the 6th and 7th categories of the Canadian Study of Health and Aging frailty scale [44]. Frailty assessed using both a frailty index and the Fried phenotype predicted mortality among community-dwelling patients with HF (mean age, 71 years) [45]. These results suggest that elderly patients with HF may require a specific functional and nutritional evaluation to allow the development of a customised treatment plan aimed at improving outcomes. A number of measures might prove useful. For example, studies in chronic HF showed that exercise training produced statistically significant improvements in self-reported health status among both younger adults [46] and elderly individuals [47], although mortality was not significantly affected. Furthermore, the programmes for elderly patients focused mainly on aerobic and resistance training and did not include a routine assessment of nutritional and functional status [47]. The cost-effectiveness of a multidisciplinary disease management programme has been evaluated in elderly outpatients with HF and mild-to-moderate frailty [48]. Interventional studies might be useful to assess whether similar programmes decrease mortality and/or improve function and quality of life of elderly patients discharged after an ADHF episode.

In a previous study, cognitive impairment was associated with higher long-term mortality in elderly patients with chronic HF, but no multivariable analysis was reported [49]. In our cohort, a crude association was observed, but only a trend towards an association persisted in the age-adjusted analysis and there was no significant association by multivariable analysis. Other prognostic factors in elderly patients with ADHF have been reported [4, 5, 7, 9–11, 17]. We found no significant associations with long-term mortality for other factors, such as diabetes, myocardial infarction, stroke, and hyponatremia. These results may be related to the inclusion of functional impairment in our multivariable model and to differences in inclusion criteria across studies.

### Strength and limitations

The inclusion in a vast multicentre cohort of unselected elderly patients with successful in-hospital treatment for ADHF and the low lost-to-follow-up rate over 2 years (7.5%) support the validity of our results. Furthermore, the inclusion of consecutive patients limited the risk of selection bias, and the main endpoint (overall mortality) is a robust criterion that leaves little room for classification bias. Finally, we routinely adjusted all analyses for age as a potential confounder, and we took interactions and confounding into account in the analyses. The similar HR values obtained after bootstrapping resampling procedures further support the robustness of our findings. Moreover, few data exist on long-term outcomes of patients older than 75 years after successful inhospital ADHF management. A limitation of our study is the absence in the analysis of several factors having previously reported associations with increased mortality, such as, ventricular function, BNP or NT-proBNP concentration, and treatments. Several studies suggest that a score including NT-proBNP may predict post-discharge 1-year mortality in ADHF populations with a mean age of about 60-75 years; therefore, in our population of very elderly patients, using this score to adjust the fragility indicators associated with post-discharge mortality would have been of interest [15]. However, our findings demonstrate the prognostic value of geriatric characteristics in elderly patients with ADHF.

## Conclusion

Independent predictors of 2-year mortality in unselected elderly patients discharged after inhospital treatment for ADHF included not only older age, male sex, lower systolic blood pressure, and lower creatinine clearance; but also geriatric markers of frailty, such as functional impairment and recent weight loss. An assessment of frailty should therefore be considered an integral part of the evaluation of elderly patients admitted for ADHF. For this frail population, an interdisciplinary approach targeting the multidimensional aspects of health may improve outcomes.

## Additional file

Additional file 1: Table S1. Factors independently associated with 1-year mortality by multivariable analysis (*n =* 399). (DOC 40 kb)

## Abbreviations

ADHF: Acutely Decompensated Heart Failure
ADL: Activities of Daily Living scale
AIC: Akaike Information Criterion
BNP: Brain Natriuretic Peptide
CI: Confidence Interval
GDS: Geriatric Depression Scale
HF: Heart Failure
HR: Hazard Ratio
MMSE: Mini Mental State Examination
MNA-SF: Mini Nutritional Assessment-Short Form
NT-proBNP: N-Terminal pro-Brain Natriuretic Peptide
NYHA: New York Heart Association classification
SD: Standard Deviation
STROBE: Strengthening the Reporting of Observational Studies in Epidemiology
