# Supplementary material for: Long-term mortality in older patients discharged after acute decompensated heart failure: a prospective cohort study
Source: BMC Geriatr. 2017 Jan 26;17:34. doi: 10.1186/s12877-017-0419-2 (PMC5270303; doi:10.1186/s12877-017-0419-2)
Supplement: Additional file 1: Table S1. — Factors independently associated with 1-year mortality by multivariable analysis (n = 399). (DOC 40 kb) [file 12877_2017_419_MOESM1_ESM.doc]

**Table S1: Factors independently associated with 1-year mortality by multivariable** analysis (n=399)

|  | Model development^a^ |  | Parameter estimates after bootstrapping methods |  |
| --- | --- | --- | --- | --- |
|  | HR 95%CI | *P* value | Mean HR 95%CI | *P* value |
| Male sex | 1.46 [1.04-2.07] | 0.03 | 1.46 [1.03-2.07] | 0.03 |
| Age >85 years | 1.58 [1.15-2.18] | <0.01 | 1.58 [1.14-2.19] | <0.01 |
| Number of impaired ADL^b^ items | 1.13 [1.05-1.20] | <0.01 | 1.13 [1.06-1.20] | <0.01 |
| Recent weight loss^c^ | 1.47 [0.98-2.20] | 0.06 | 1.47 [0.95-2.26] | 0.08 |
| Systolic blood pressure (mmHg)^d^ | 0.82 [0.70-0.98] | 0.02 | 0.85 [0.68-0.99] | 0.04 |
| Creatinine clearance ≤30 mL/minute^e^ | 1.34 [0.90-2.16] | 0.14 | 1.40 [0.89-2.18] | 0.14 |

HR, hazards ratio; CI, confidence interval; ADL, activities of daily living scale

^a^ Hazards ratios and confidence intervals were estimated using Cox proportional models simultaneously adjusted for all variables listed in the table.

^b^ per additional impaired ADL item

^c^ >3 Kg within the 3 months preceding admission

^d^ per increase by 1 standard deviation

^e^Creatinine clearance was calculated using the abbreviated Modification of Diet in Renal Disease formula, glomerular filtration rate (mL/min/1.73 m^2^) = 186.3 × [creatinine (μmol/L) /88.4] ^-1.154^ × [Age(years)]^-0.203^ × 0.742 (if female) × 1.21 (if black)
